# Limits of Cation Solubility in *A*Mg_2_Sb_2_ (*A* = Mg, Ca, Sr, Ba) Alloys

**DOI:** 10.3390/ma12040586

**Published:** 2019-02-15

**Authors:** Wanyue Peng, Alexandra Zevalkink

**Affiliations:** Chemical Engineering and Materials Science, Michigan State University, East Lansing, MI 48824, USA; pengwany@msu.edu

**Keywords:** solubility, Zintl phases, alloy, thermoelectric

## Abstract

$AM_{2}X_{2}$ compounds that crystallize in the CaAl${}_{2}$Si${}_{2}$ structure type have emerged as a promising class of *n*- and *p*-type thermoelectric materials. Alloying on the cation (*A*) site is a frequently used approach to optimize the thermoelectric transport properties of $AM_{2}X_{2}$ compounds, and complete solid solubility has been reported for many combinations of cations. In the present study, we investigate the phase stability of the *A*Mg${}_{2}$Sb${}_{2}$ system with mixed occupancy of Mg, Ca, Sr, or Ba on the cation (*A*) site. We show that the small ionic radius of Mg${}^{2+}$ leads to limited solubility when alloyed with larger cations such as Sr or Ba. Phase separation observed in such cases indicates a eutectic-like phase diagram. By combining these results with prior alloying studies, we establish an upper limit for cation radius mismatch in $AM_{2}X_{2}$ alloys to provide general guidance for future alloying and doping studies.

## 1. Introduction

**Figure 1:**
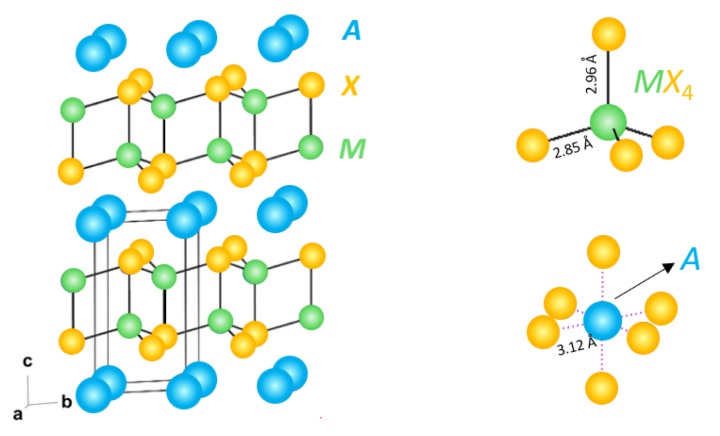
The crystal structure of $AM_{2}X_{2}$ compounds with CaAl${}_{2}$Si${}_{2}$ structure type, characterized by anionic (M${}_{2}$X${}_{2}$)${}^{2-}$ slabs sandwiched by monolayers of A${}^{2+}$ cations. In the present study, we investigate alloying of *A* = Mg, Ca, Sr, and Ba in *A*Mg${}_{2}$Sb${}_{2}$. The bond distances labeled are that of MgMg${}_{2}$Sb${}_{2}$.

$AM_{2}X_{2}$ compounds with the CaAl${}_{2}$Si${}_{2}$ structure type (space group $P\bar{3}m1$) are a promising emerging class of thermoelectric materials, with zT values up to 1.2 and 1.6 for *p*-type [1] and *n*-type [2,3,4,5,6] samples, respectively, at intermediate temperatures. Within this structure type, the compounds with electronic properties most suitable for thermoelectric applications (e.g., those with narrow band gaps) contain *A* = a divalent alkali or alkaline earth or rare earth metal, *M* = a divalent metal (e.g., Zn, Cd, Mn, or Mg), and *X* = a group 15 element [7]. The structure is characterized by anionic (M${}_{2}$X${}_{2}$)${}^{2-}$ slabs sandwiched by monolayers of $A^{2+}$ cations [8], as shown in Figure 1.

In recent years, the binary compound MgMg${}_{2}$Sb${}_{2}$, in which Mg occupies both the tetrahedrally coordinated *M* site and the octahedrally coordinated *A* site, has attracted a great deal of attention. Unlike other compounds with this structure type, MgMg${}_{2}$Sb${}_{2}$ can be successfully *n*-type doped, leading to the highest thermoelectric figure of merit, zT = 1.6, among $AM_{2}X_{2}$ compounds to date [2,4,9,10,11]. Since then, various studies have explored dopability [12,13], band engineering [3,5,14,15], and the origins of the inherently low lattice thermal conductivity [16,17,18] in MgMg${}_{2}$Sb${}_{2}$ and MgMg${}_{2}$Bi${}_{2}$.

Thus far, much of the optimization of *n*-type MgMg${}_{2}$Sb${}_{2}$ has focused on alloying or doping on the anion site (e.g., Te doping on the anion site of MgMg${}_{2}$(Sb,Bi)${}_{2}$). In contrast, alloying of two or more elements on the cation site (*A* = Mg, Ca, Sr, Ba, Sm, Eu, Yb) was frequently used in earlier work on *p*-type $AM_{2}X_{2}$ compounds to improve and optimize thermoelectric performance. For example, mixed occupancy of *A* = Ca, Yb, and Eu in the *A*Zn${}_{2}$Sb${}_{2}$ [19,20,21] and *A*Cd${}_{2}$Sb${}_{2}$ systems [20,22,23,24], and more recently in the *A*Mg${}_{2}Pn_{2}$ system, have been investigated [15,25,26]. Alloying on the cation site has two main benefits: first, the site disorder often improves the ratio of electronic mobility to lattice thermal conductivity [21,22,26]. Equally importantly, alloying subtly tunes the defect formation energy for cation vacancies—the dominant intrinsic defect in this structure type—which allows for optimization of the carrier concentration without introducing additional dopants [27,28]. Although isovalent alloying on the cation site in *n*-type MgMg${}_{2}Pn_{2}$ has not yet been reported, one would expect that similar zT enhancements could be achieved through this strategy (assuming that *n*-type doping can still be achieved for *A*≠ Mg.)

To date, complete solubility has been reported in most investigations of alloys between different $AM_{2}X_{2}$ compounds in the CaAl${}_{2}$Si${}_{2}$ structure type, regardless of whether the substitutions were made on the *A*, *M* or *X* site [19,20,21,22,23,24,25,26]. In the present study, we probe the solubility limits of larger cations (e.g., Ca, Sr, Ba) alloyed with Mg on the *A* cation site in *A*Mg${}_{2}$Sb${}_{2}$ compounds. Even though alloying on the *A* site seems to be an applicable approach to further optimize the thermoelectric properties of n-type MgMg${}_{2}$Sb${}_{2}$, the present study shows that the small ionic radius of Mg${}^{2+}$ leads to limited solubility of any cation larger than Ca. We use the present investigation to develop general predictions for cation solubility in $AM_{2}X_{2}$ compounds, which may provide guidance for future work on this class of material.

## 2. Methods

### 2.1. Synthesis

Samples of (Ca${}_{x}$Mg${}_{1-x}$)Mg${}_{2}$Sb${}_{2}$ (*x* = 0, 0.1, 0.2, 0.3, 0.4, 0.5, 0.6, 0.7, 0.8, 1), (Sr${}_{x}$Mg${}_{1-x}$)Mg${}_{2}$Sb${}_{2}$ (*x* = 0, 0.1, 0.2, 0.4, 0.6, 0.7, 0.8, 0.9, 1), (Ba${}_{x}$Mg${}_{1-x}$)Mg${}_{2}$Sb${}_{2}$ (*x* = 0, 0.3, 0.5, 0.8, 0.9, 1), and (Ba${}_{x}$Ca${}_{1-x}$)Mg${}_{2}$Sb${}_{2}$ (*x* = 0, 0.1, 0.3, 0.5, 0.7, 0.9, 1) were synthesized via direct ball-milling of elements followed by spark plasma sintering (SPS). The corresponding stoichiometric elements (99.8% Mg shot, 99.5% Ca shot, 99% Sr chunk and 99%+ Ba rod, and 99.99% Sb from Alfa Aesar) were cut into small pieces in an argon filled glove box, loaded into stainless steel vials with two stainless steel balls, and milled for one hour using a SPEX mill, SPEX SamplePrep LLC., Metuchen, NJ, USA. The ball-milled powder was then loaded into graphite dies with 10 mm inner diameter. The powder was heated to the target temperatures shown in Table 1 in 10 min, and held at that temperature for 10 min under a pressure of 31 MPa using a DR. Sinter SPS-211LX, Fuji Electronic Industrial Co., LTD, Tsurugashima, Japan. The SPS temperatures of the alloyed samples were between the synthesis temperature of the two pure compounds. The exact temperatures chosen were determined by the sample density and quality after observing the X-ray diffraction pattern. The pressure was removed immediately when cooling started. The densities of all the samples were obtained by measurement of mass and geometry, yielding at least 97% of the theoretical density.

All of the alloyed samples were annealed after SPSing to ensure homogeneity. The samples were wrapped in graphite foils and sealed in glass ampules under vacuum, which were then held at 500 ${}^{\circ }C$ for ten days. The samples were quenched to room temperature in air. X-ray diffraction was performed before and after annealing.

### 2.2. Structural Characterization

X-ray diffraction was performed on bulk samples using a Rigaku Smartlab X-ray diffraction system with Cu K-α radiation to identify the compositions. Phase purity of the samples was confirmed via peak matching within the ICSD database. Lattice parameters were obtained via Rietveld refinement using the PDXL2 software (version 2) for samples both before and after annealing. The values and uncertainties of the lattice parameters are included in Supplemental Tables S1–S4.

## 3. Results and Discussion

Among *A*Mg${}_{2}$Sb${}_{2}$ and *A*Mg${}_{2}$Bi${}_{2}$ compounds, Mg is the smallest cation that can occupy the octahedral site (*A*). The ionic radius of Mg${}^{2+}$ in an octahedral environment is 0.72 Å, which is significantly smaller than that of Ca${}^{2+}$ (1.00 Å), Sr${}^{2+}$ (1.18 Å) or Ba${}^{2+}$ (1.35 Å) [29,30]. The divalent rare-earth metals Sm, Eu, and Yb can also occupy the cation site, with ionic radii in between that of Ca and Sr. In the present study, alloyed samples in the series (Ca${}_{x}$Mg${}_{1-x}$)Mg${}_{2}$Sb${}_{2}$, (Sr${}_{x}$Mg${}_{1-x}$)Mg${}_{2}$Sb${}_{2}$, (Ba${}_{x}$Mg${}_{1-x}$)Mg${}_{2}$Sb${}_{2}$ were synthesized to investigate the phase stability when cations of increasingly divergent ionic radii occupy the *A* site in *A*Mg${}_{2}$Sb${}_{2}$. Note that Ca, Sr, and Ba are believed to exclusively occupy the *A* site, being too large to occupy the tetrahedrally coordinated *M* site in $AM_{2}X_{2}$ compounds. Thus, with increasing Ca, Sr, or Ba content, we expect to observe mixed occupancy on the *A* = Mg site only, not on the *M* = Mg site.

**Figure 2:**
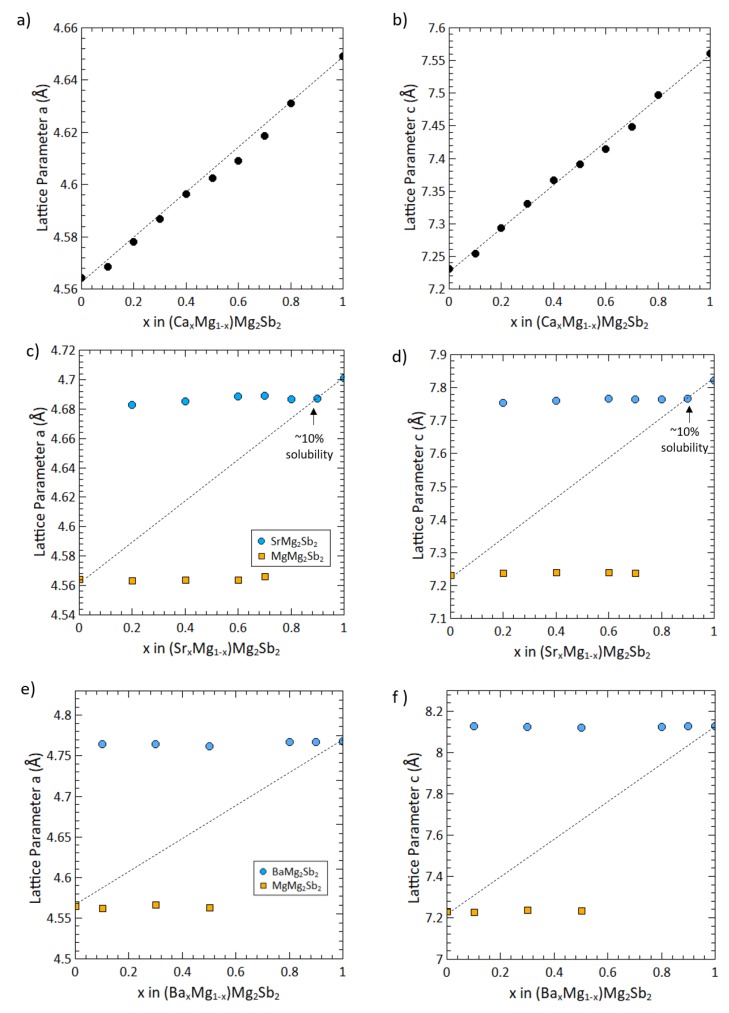
(**a**,**b**) For the (Ca${}_{x}$Mg${}_{1-x}$)Mg${}_{2}$Sb${}_{2}$ series, the lattice parameters *a* and *c* underwent a linear change with calcium alloying ratio. (**c**,**d**) For the (Sr${}_{x}$Mg${}_{1-x}$)Mg${}_{2}$Sb${}_{2}$ series, the lattice parameters indicate a 10% solubility for Sr on Mg site, and no solubility for Mg on Sr site. (**e**,**f**) For (Ba${}_{x}$Mg${}_{1-x}$)Mg${}_{2}$Sb${}_{2}$ series, the lattice parameters show no solubility between Ba and Mg on the cation site. Please note that the MgMg${}_{2}$Sb${}_{2}$ phase can be observed from X-ray diffraction pattern when *x* = 0.8 in the Sr-Mg series and for *x* = 0.8 and 0.9 in the Ba-Mg series, but the peak intensities are too low for reliable refinement of lattice parameters.

For the (Ca${}_{x}$Mg${}_{1-x}$)Mg${}_{2}$Sb${}_{2}$ series, we find that the lattice parameters undergo a linear change with calcium alloying for *x* = 0–1 (Figure 2a,b), showing that (Ca${}_{x}$Mg${}_{1-x}$)Mg${}_{2}$Sb${}_{2}$ forms a complete solid solution according to Vegard’s rule [31]. In contrast, alloying Mg with larger cations (Sr or Ba) leads to phase separation into a Mg-rich phase and Mg-poor phase, indicative of a eutectic-like phase diagram. The lattice parameters of (Sr${}_{x}$Mg${}_{1-x}$)Mg${}_{2}$Sb${}_{2}$ and (Ba${}_{x}$Mg${}_{1-x}$)Mg${}_{2}$Sb${}_{2}$ are shown in Figure 2c–f. For the Sr-Mg alloy, a slight decrease in the lattice parameters *a* and *c* indicates a small (roughly 10%) solubility for Mg on the Sr site, but no solubility of Sr on the Mg site. In the Ba-Mg alloy, no solubility of Ba on the Mg site, or of Mg on the Ba site was observed. Please note that the lattice parameters shown in Figure 2 were measured after annealing at 500 ${}^{\circ }C$ for ten days. The lattice parameters of the samples immediately after SPSing are shown in Supplemental Tables S1–S4. However, we did not observe any obvious change in the solubility before and after annealing.

**Figure 3:**
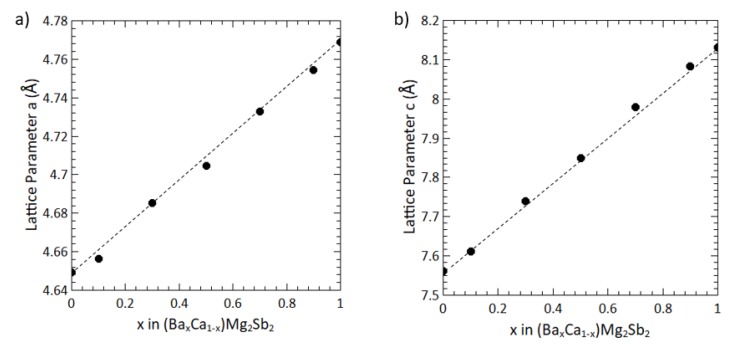
The linearly varying lattice parameters in the (Ba${}_{x}$Ca${}_{1-x}$)Mg${}_{2}$Sb${}_{2}$ alloy indicate complete solid solubility of this system. (**a**) The lattice parameters *a* and (**b**) *c* of (Ba${}_{x}$Ca${}_{1-x}$)Mg${}_{2}$Sb${}_{2}$.

In the ionic metal model proposed by Hume-Rothery for substitutional solid solutions, differences in ionic radius, polarizability, structure, valence, and electronegativity are the key factors affecting the solubility [32,33,34]. Here, the atomic size difference is expected to play a dominant role in the stability [34,35]. To estimate an upper limit for size mismatch on the cation site in the *A*Mg${}_{2}$Sb${}_{2}$ compounds discussed here, we use the limit established by the partial solubility of the Sr-Mg system. For substitutions of a small cation by a larger one, the upper limit size mismatch is estimated by ($r_{Sr}$-$r_{Mg}$)/$r_{Mg}$ = 64%. For substitutions of a larger cation by a smaller one (e.g., Mg on the Sr site) the limit is given by ($r_{Sr}$-$r_{Mg}$)/$r_{Sr}$ = 39%. To test these limits, the Ba${}_{x}$Ca${}_{1-x}$Mg${}_{2}$Sb${}_{2}$ series was synthesized. The radii difference of Ba to Ca is 35% and Ba to Ca is 25.9%, both of which are smaller than the critical size difference. As shown by the linearly increasing lattice parameters in Figure 3, the Ba${}_{x}$Ca${}_{1-x}$Mg${}_{2}$Sb${}_{2}$ alloy is found to be a complete solid solution, as predicted.

**Figure 4:**
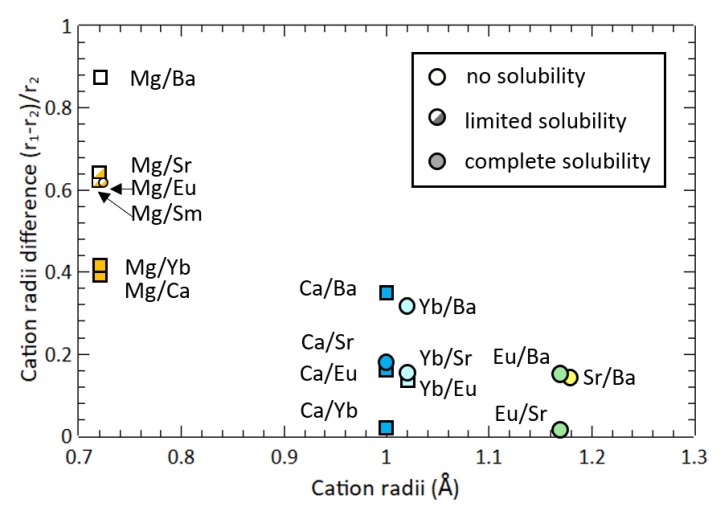
The cation radii difference of $AM_{2}X_{2}$ calculated from ($r_{1}$-$r_{2}$)/$r_{2}$ for all possible combinations of cations where $r_{1}>r_{2}$. In each combination, the smaller species is listed first. Square symbols represent experimental observations, while circles represent predictions. The limited solubility in Sr/Mg (present study) and Sm/Mg (Reference [36]) alloys provide an approximate upper limit for cation radii mismatch. The complete solubility of Ca/Eu [19,20,24], Ca/Yb [20,21,22,26], and Yb/Eu [20,23] Mg/Yb [25] have been confirmed by prior studies.

A survey of prior alloying studies suggests that the limit proposed here is generalizable to cation-site alloying for most $AM_{2}Pn_{2}$ compounds with the same structure type. Figure 4 shows the ionic radius ratio, ($r_{1}$-$r_{2}$)/$r_{2}$, for all possible combinations of cations where $r_{1}>r_{2}$. The ionic radii were obtained from ref [29,30] using the values for 2+ valence and 6-fold coordination. Square symbols indicate cation combinations that have been experimentally attempted, while circles represent our predictions. Indeed, this figure illustrates that it is only possible to exceed the predicted size mismatch limit by alloying with Mg on the cation site. All other combinations have sufficiently similar ionic radii (e.g., Ca-Yb [20,21,22,26],Ca-Eu [19,20,24], Yb-Eu [20,23], Mg-Yb [25]) to form complete solid solutions.

One notable exception in the literature is the (Sm,Mg)Mg${}_{2}$Sb${}_{2}$ system, investigated in 2006 by Gupta et al. [36]. Depending on synthesis conditions, alloying with Sm on the Mg site was shown to lead to either phase separation or to the formation of a superstructure in which Mg and Sm occupy alternating cation monolayers. The ionic radii of Sm${}^{2+}$ is similar to that of Sr${}^{2+}$, which appears to be around the upper limit for ionic radius on the *A* = Mg site. However, no superstructure formation was observed after quenching from high temperature for any of our alloyed samples. The upper size limit established here may provide guidance for doping on the Mg site in MgMg${}_{2}$Sb${}_{2}$. Recently, La${}^{3+}$ and Y${}^{3+}$ on the Mg site were successfully used as *n*-type dopants [12,37]. The ionic radii of La${}^{3+}$ are similar to that of Yb${}^{2+}$, as are the radii of the majority of trivalent lanthanides, and Y${}^{3+}$ has a even smaller radii between Yb${}^{2+}$ and Mg${}^{2+}$. This suggests that the radii of these *n*-type dopants will not be a primary factor limiting their solubility. We note, however, that complete solid solubility would never be expected for alio-valent dopants in Zintl phases. Furthermore, in alio-valent doping, the size of the dopant is only a minor factor. Other factors controlling solubility include the valence of the dopant and the impact that the dopant has on the chemical potential of other types of defects.

## 4. Conclusion

In $AM_{2}X_{2}$ alloys, the existence of complete solid solubility is found to depend strongly on the difference between the ionic radii of the alloyed species. For mixed occupancy on the cation site, the partial solubility of Mg on the Sr site in the (Mg${}_{x}$Sr${}_{1-x}$)Mg${}_{2}$Sb${}_{2}$ series indicates that the size mismatch of Sr and Mg can be used as an approximate upper limit to guide future alloying and doping studies. Indeed, among all cations that are known to occupy the *A* site in $AM_{2}X_{2}$ compounds, we find that only Mg is sufficiently small to lead to phase separation, and only when alloyed with cations with radii equal to or larger than Sr (e.g., Ba, Eu, or Sm).

## Figures and Tables

**Table 1:** The SPS temperatures of (Ca${}_{x}$Mg${}_{1-x}$)Mg${}_{2}$Sb${}_{2}$, (Sr${}_{x}$Mg${}_{1-x}$)Mg${}_{2}$Sb${}_{2}$, (Ba${}_{x}$Mg${}_{1-x}$)Mg${}_{2}$Sb${}_{2}$, and (Ba${}_{x}$Ca${}_{1-x}$)Mg${}_{2}$Sb${}_{2}$.

(Ca${}_{x}$Mg${}_{1-x}$)Mg${}_{2}$Sb${}_{2}$	*x* = 0	*x* = 0.1	*x* = 0.2	*x* = 0.3	*x* = 0.4	*x* = 0.5	*x* = 0.6	*x* = 0.7	*x* = 0.8	*x* = 1
Temperature (${}^{\circ }C$)	850	810	790	770	750	730	710	690	670	650
(Sr${}_{x}$Mg${}_{1-x}$)Mg${}_{2}$Sb${}_{2}$	*x* = 0	*x* = 0.1	*x* = 0.2	*x* = 0.4	*x* = 0.6	*x* = 0.7	*x* = 0.8	*x* = 0.9	*x* = 1	-
Temperature (${}^{\circ }C$)	850	800	750	750	750	750	750	750	700	-
(Ba${}_{x}$Mg${}_{1-x}$)Mg${}_{2}$Sb${}_{2}$	*x* = 0	*x* = 0.3	*x* = 0.5	*x* = 0.8	*x* = 0.9	*x* = 1	-	-	-	-
Temperature (${}^{\circ }C$)	850	750	700	700	700	700	-	-	-	-
(Ba${}_{x}$Ca${}_{1-x}$)Mg${}_{2}$Sb${}_{2}$	*x* = 0	*x* = 0.1	*x* = 0.3	*x* = 0.5	*x* = 0.7	*x* = 0.9	*x* = 1	-	-	-
Temperature (${}^{\circ }C$)	650	700	700	700	700	700	700	-	-	-

## Supplementary Materials

The following are available online at http://www.mdpi.com/1996-1944/12/4/586/s1, Tables S1–S4: Lattice parameters and R${}_{wp}$ values from Reitveld refinements. Figure S1: Reitveld refinement for the (Ca${}_{0.7}$Mg${}_{0.3}$)Mg${}_{2}$Sb${}_{2}$ sample is representative of data for single-phase samples in the Ca-Mg and Ba-Ca solid solution series. Figure S2: (Ca${}_{0.7}$Mg${}_{0.3}$)Mg${}_{2}$Sb${}_{2}$ sample is representative of data for single-phase samples in the Ca-Mg and Ba-Ca solid solution series. Figure S3: Rietveld refinement for the (Sr${}_{0.4}$Mg${}_{0.6}$)Mg${}_{2}$Sb${}_{2}$ sample is representative of data for two-phase samples in the Sr-Mg and Ba-Mg series. Note that the C-graphite peak is from some residual graphite foil on the surface and edges of the sample (not in the sample). Figure S4: Rietveld refinement for the (Sr${}_{0.4}$Mg${}_{0.6}$)Mg${}_{2}$Sb${}_{2}$ sample is representative of data for two-phase samples in the Sr-Mg and Ba-Mg series. Note that the C-graphite peak is from some residual graphite foil on the surface and edges of the sample (not in the sample).
