# Supplementary material for: Limits of Cation Solubility in AMg2Sb2 (A = Mg, Ca, Sr, Ba) Alloys
Source: Materials (Basel). 2019 Feb 15;12(4):586. doi: 10.3390/ma12040586 (PMC6416610; doi:10.3390/ma12040586)
Supplement: Supplementary file 1 [file materials-12-00586-s001.pdf]

Limits of cation solubility in $A\mathrm{Mg_2Sb_2}$ (A=Mg, Ca, Sr, Ba) alloys

1 Lattice parameter refinement

Table S1: Lattice parameters and R_{wp} values from powder XRD of the reitveld refinements of $(Ca_xMg_{1-x})Mg_2Sb_2$.

	After SPS			After annealing 10 days		
x	a	c	R_{wp}	a	c	R_{wp}
0	4.56436(14)	7.2310(2)	3.66	-	-	-
0.1	4.5760(2)	7.2631(2)	3.54	4.56854(18)	7.25428(17)	3.85
0.2	4.5856(2)	7.3033(4)	3.86	4.57815(16)	7.2932(2)	3.7
0.3	4.58928(15)	7.3319(2)	2.89	4.58691(11)	7.3304(2)	3.16
0.4	4.59639(11)	7.36216(17)	3.38	4.59622(11)	7.36684(17)	3.33
0.5	4.60429(12)	7.3853(2)	2.62	4.60254(11)	7.3905(18)	3.1
0.6	4.61042(9)	7.41467(14)	2.72	4.60919(11)	7.41438(18)	2.71
0.7	4.62359(10)	7.45927(17)	2.12	4.61850(7)	7.44851(13)	2.21
0.8	4.62980(7)	7.49189(14)	2.74	4.63103(16)	7.4971(2)	2.34
0.9	4.64026(8)	7.52549(14)	1.98	-	-	-
1	4.64913(18)	7.56089(2)	2.42	-	-	-

Table S2: Lattice parameters and R_{wp} values from powder XRD of the refinements of $(Sr_xMg_{1-x})Mg_2Sb_2$. With the exception of the $X = 0.9$ sample, all alloyed samples contain both a Sr-rich and a Mg-rich phase.

	After SPS					After annealing 10 days				
	Sr-rich phase		Mg-rich phase			Sr-rich phase		Mg-rich phase		
x	a	c	a	c	R_{wp}	a	c	a	c	R_{wp}
0	-	-	4.56436 (14)	7.2310 (2)	3.66	-	-	-	-	-
0.2	4.6841 (2)	7.7482 (7)	4.57706 (10)	7.2805 (2)	2.9	4.6827(4)	7.7533(8)	4.56333(17)	7.2389(3)	2.28
0.4	4.6850 (3)	7.7570 (5)	4.5736 (3)	7.2721 (15)	2.19	4.6852(2)	7.7594(4)	4.5639(3)	7.2406(4)	2.92
0.6	4.68527 (19)	7.7568 (3)	4.5711 (4)	7.2599 (8)	1.9	4.68857(11)	7.7671(19)	4.5637(5)	7.2407(8)	1.81
0.7	4.68489 (17)	7.7597 (3)	4.5694 (6)	7.2643 (10)	1.83	4.6891(9)	7.765(15)	4.5664(2)	7.2377(7)	1.83
0.8	4.69262 (8)	7.78273 (17)	-	-	2.18	4.68644(19)	7.7651(3)	-	-	2.03
0.9	4.68927 (12)	7.7681 (2)	-	-	2.47	4.68727(9)	7.76557(16)	-	-	-
1	4.70068 (7)	7.82196(13)	-	-	1.59	-	-	-	-	-

Table S3: Lattice parameters and R_{wp} values from powder XRD of the refinements of $(Ba_xMg_{1-x})Mg_2Sb_2$. All alloyed samples separated into a Ba-rich and a Mg-rich phase, suggesting zero solubility.

	After SPS					After annealing 10 days				
	Ba-rich phase		Mg-rich phase			Ba-rich phase		Mg-rich phase		
x	a	c	a	c	R_{wp}	a	c	a	c	R_{wp}
0	-	-	4.56436 (14)	7.2310 (2)	3.66	-	-	-	-	-
0.1	4.7615 (3)	8.1174 (8)	4.5649 (15)	7.2327 (18)	3.06	4.7647 (3)	8.1269 (10)	4.56246 (15)	7.22828 (19)	2.85
0.3	4.7645 (3)	8.1252 (5)	4.5653 (4)	7.2335 (5)	2.71	4.7642 (4)	8.1249 (8)	4.56622 (18)	7.2372 (3)	2.73
0.5	4.7716 (3)	8.1319 (2)	4.5682 (4)	7.2302 (9)	2.83	4.76237 (14)	8.1222 (3)	4.56307 (17)	7.2346 (4)	2.47
0.8	4.76800 (15)	8.1250 (3)	4.564 (4)	7.1924 (8)	3.64	4.7665 (3)	8.1236 (4)	-	-	3.31
0.9	4.7624 (18)	8.122 (3)	4.5746 (17)	7.2295 (5)	3.19	4.76172 (18)	8.1196 (3)	-	-	3.8
1	4.76767 (15)	8.1293 (3)	-	-	2.82	-	-	-	-	-

Table S4: Lattice parameters and R_{wp} values from powder XRD of the refinements of $(Ba_xCa_{1-x})Mg_2Sb_2$.

	After SPS			After annealing 10 days		
x	a	c	R_{wp}	a	c	R_{wp}
0	4.64913(18)	7.56089(2)	3.66	-	-	-
0.1	4.65967(10)	7.61872(15)	2.99	4.6563(3)	7.6121(4)	2.22
0.3	4.6821(2)	7.7356(4)	4.33	4.68539(15)	7.740300(3)	3.56
0.5	4.7077(10)	7.8544(16)	5.97	4.7046(2)	7.8483(4)	3.46
0.7	4.7328(2)	7.9790(4)	4.64	4.7328(2)	7.9790(5)	1.83
0.9	4.76027(14)	8.0907(3)	2.81	4.75453(16)	8.0838(3)	3.73
1	4.76767(15)	8.1293(3)	2.82	-	-	-

2 Representative Rietveld refinements

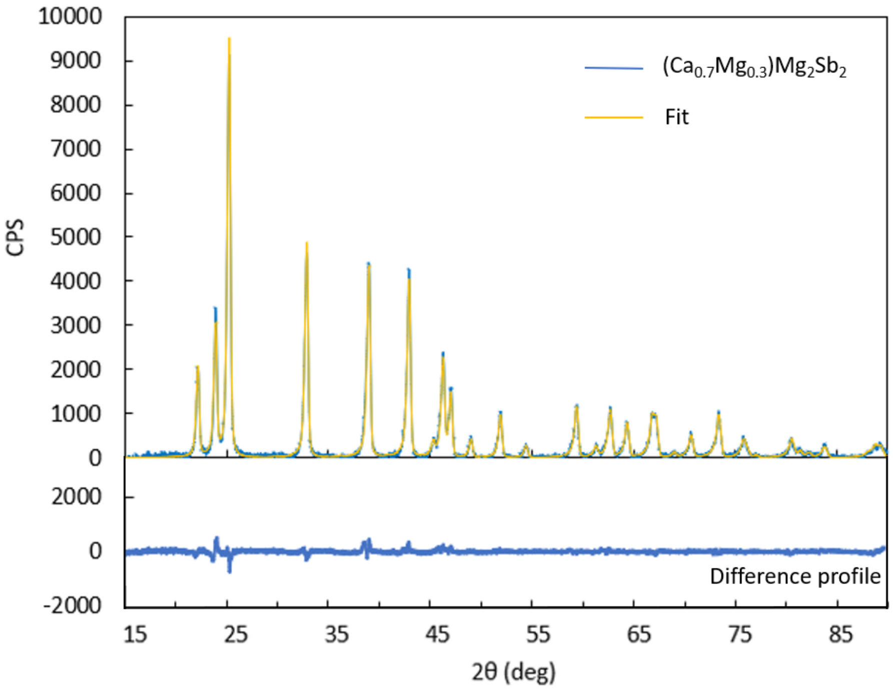

Figure S1: Rietveld refinement for the $(Ca_{0.7}Mg_{0.3})Mg_2Sb_2$ sample is representative of data for single-phase samples in the Ca-Mg and Ba-Ca solid solution series.

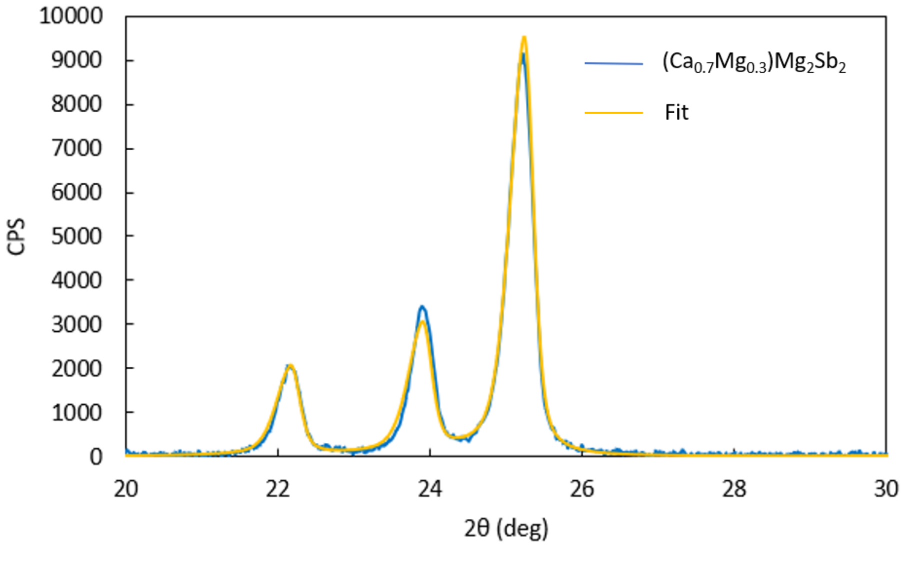

Figure S2: Rietveld refinement for the $(Ca_{0.7}Mg_{0.3})Mg_2Sb_2$ sample is representative of data for single-phase samples in the Ca-Mg and Ba-Ca solid solution series.

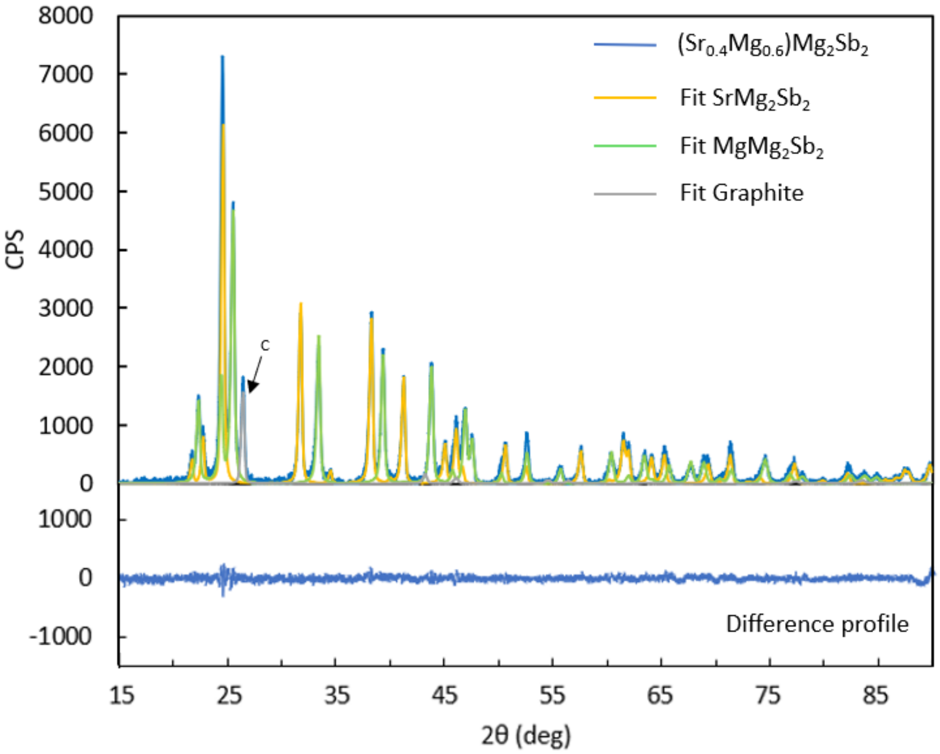

Figure S3: Rietveld refinement for the $(Sr_{0.4}Mg_{0.6})Mg_2Sb_2$ sample is representative of data for two-phase samples in the Sr-Mg and Ba-Mg series. Note that the C-graphite peak is from some residual graphite foil on the surface and edges of the sample (not in the sample).

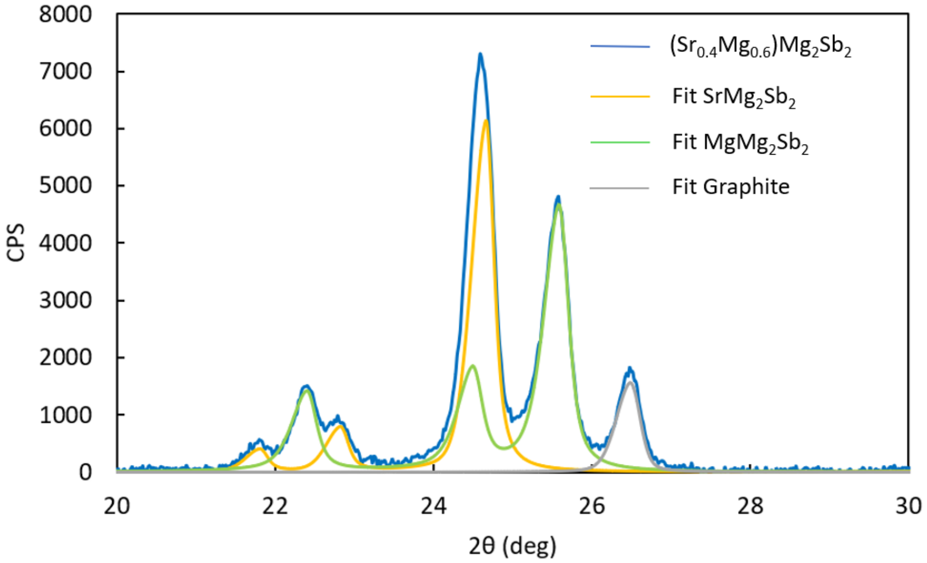

Figure S4: Rietveld refinement for the $(Sr_{0.4}Mg_{0.6})Mg_2Sb_2$ sample is representative of data for two-phase samples in the Sr-Mg and Ba-Mg series. Note that the C-graphite peak is from some residual graphite foil on the surface and edges of the sample (not in the sample).
